# Effect of Train-Induced Wind on the Transmission of COVID-19: A New Insight into Potential Infectious Risks

**DOI:** 10.3390/ijerph18158164

**Published:** 2021-08-02

**Authors:** Simin Zou, Xuhui He

**Affiliations:** 1 School of Civil Engineering, Central South University, Changsha 410075, China; 2 Natiaonl Engineering Laboratory for High Speed Railway Construction, Changsha 410075, China; 3 Joint International Resarch Laboratory of Key Technology for Rail Traffic Safety, Changsha 410075, China; 4 Hunan Provincial Key Laboratory for Disaster Prevention and Mitigation of Rail Transit Engineering Structure, Changsha 410075, China

**Keywords:** high-speed train, train-induced wind, COVID-19, moving model test, Gaussian puff model

## Abstract

The unprecedented COVID-19 pandemic has caused a traffic tie-up across the world. In addition to home quarantine orders and travel bans, the social distance guideline of about six feet was enacted to reduce the risk of contagion. However, with recent life gradually returning to normal, the crisis is not over. In this research, a moving train test and a Gaussian puff model were employed to investigate the impact of wind raised by a train running on the transmission and dispersion of SARS-CoV-2 from infected individuals. Our findings suggest that the 2 m social distance guideline may not be enough; under train-induced wind action, human respiratory disease-carrier droplets may travel to unexpected places. However, there are deficiencies in passenger safety guidelines and it is necessary to improve the quantitative research in the relationship between train-induced wind and virus transmission. All these findings could provide a fresh insight to contain the spread of COVID-19 and provide a basis for preventing and controlling the pandemic virus, and probe into strategies for control of the disease in the future.

## 1. Introduction

Coronavirus disease 2019 (COVID-19) is a respiratory tract infection caused by SARS-CoV-2. Since the novel coronavirus disease 2019 (COVID-19) discovered at the end of 2019, an unprecedented pandemic has created a global public health threat. As of May 2021, the worldwide coronavirus disease epidemic brought the cumulative numbers to over 169 million reported cases, and more than 3.5 million lives were taken [1]. Recent clinical studies have demonstrated that COVID-19 is gradually becoming one of the major causes of death in some countries, such as the USA and Belgium, for 2020 [2]. Because the virus proved to have a horrifying destructive power and was highly contagious, the rate of patient growth has still remained stubbornly high [3]. Worse still, different than most other respiratory illness such as the influenza virus, SARS-CoV-2 may seem out of control, growing wildly and breaking all the rules and routines. Although COVID-19 is not an incurable disease and taking some measures (e.g., home quarantine, wearing masks) may provide short-term relief from infectious diseases, recent alarming results from a new study say that a large percentage of patients who have recovered from COVID-19 still could be potential virus carriers [4].

COVID-19 has influenced human society on all aspects and levels, and all have suffered grievously. The social and economic environment has a direct influence on COVID-19 transmission [5]; additionally, the reach of COVID-19 has been omni-directional, with lasting reactions on social and economic development, not just the human body [6,7]. With the end of social confinement, the economic circles and social activities which had shut down for a while have subsequently restarted, and the COVID-19 emergence risk is resurfacing in current life [8]. Many countries have halted some or all travel since the outbreak of the COVID-19 pandemic. Nevertheless, now the resumption of work after the suspension is underway, and plans to re-open travel are being formed.

**Figure 1:**
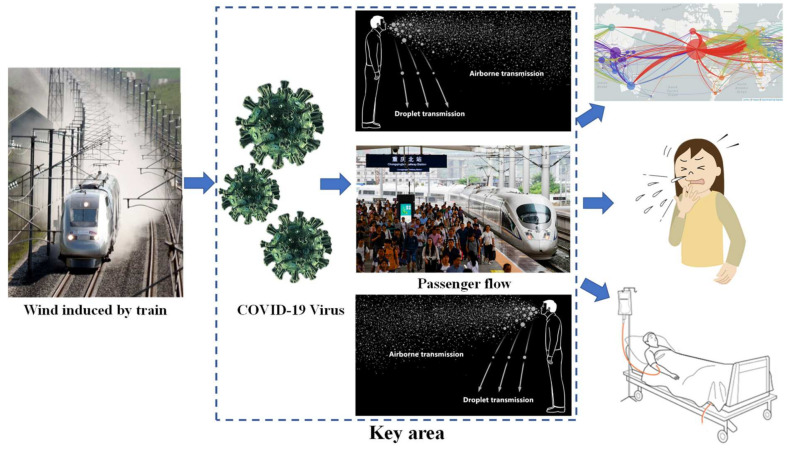
COVID-19 model of the airborne (wind-spread) transmission pathway near the train.

Railway transportation is a relatively economical, convenient, and safe way to travel, and is the busiest form of transportation, carrying millions of passengers each day, especially in China, Japan, Europe, and India [9]. A high-speed train is a standard traffic tool that needs to meet the requirement of transport services, which is inextricably linked to people’s lives as the number of passengers has grown and become much closer both in intra-regional and inter-regional economy and trade. In this case, sudden, massive, and dispersive human migration can extend localized onsets of disease into extensive epidemics [10]. The railway station, where crowds congregate, may become a place similar to a virus petri dish when a quarantinable infectious disease is prevalent, as shown in Figure 1. It is important to notice that the infective dose needed to become diseased with COVID-19 is still unknown so far. The study in [11] showed that crowd density and wind have a strong influence on spreading the virus spread. The relationship between wind speed and the virus spread were totally mediated by the crowd density [12]. Furthermore, infants, young children, and immunocompromised people may be infectious for even longer as differences exist in infectious SARS-CoV-2 between individuals. According to a recent study from [13], an immunocompromised patient infected with SARS-CoV-2 for at least 105 days (no symptoms) and at least 70 days of detoxification was potentially infectious. Therefore, in this situation, the glaring issue here is the effect of train-induced wind on the virus transmission from the possible infected individual, as shown in Figure 1. 

COVID-19 has already transformed our lives and society. SARS-CoV-2 can stay in the atmosphere for a long time and continue to reverberate through the world. However, as transportation is an important component of necessity in social lives, the monitoring of public traffic environments actively to identify and address potential problems becomes urgent in the current outbreak of the virus in human society. This study outlines vital considerations for the potential influence of train-induced wind on virus transmission. Using a new moving train model, a test rig was developed in the Central South University to acquire in-depth knowledge of train-induced wind with a new China Railway High-speed (CRH) train model. Subsequently, the effect of train-induced wind on the virus transmission was investigated in terms of virus concentration using the obtained experimental results combined with the Gaussian puff model. Subsequently, some conclusions formulated from the experimental results and theoretical analysis are presented to visualize and introduce the study’s vision about the employee working and passenger waiting environment. Despite some countermeasures taken to ensure urban residents’ health and hygiene, the public health effort is still vulnerable. This study strives to investigate appropriate physical defenses, behavior rules, planning theories, and regulations to contribute the indispensable protection from virus attacks and also strives to continue to strengthen our living environmental defence. Therefore, this study aims to explore the effect of train-induced wind on the COVID-19 pandemic. This study attempt to illustrate the role of train-induced wind on COVID-19 transmission for the first time. This paper offers a new insight into the spread of COVID-19 as well as other infectious respiratory diseases.

## 2. Background

### 2.1. Survey on the Transmission of COVID-19

SARS-CoV-2 is highly uncertain and intricate in space and time [14]. SARS-CoV-2 today seems to be less environmentally restricted than ever before. In this regard, the virus with strains from previous outbreaks was considered relatively sensitive to temperature, season, and climate [15,16,17]. Thus, a long standing suppostition is that the low temperatures and low humidities prevalent in the dry and cold season somehow enhance influenza virus transmission [18]. In recent studies conducted to understand the relationship between environment and COVID-19 cases, extremely unusual results were shown [19,20,21]. The exact nature of the relationship of underlying environmental factors to SARS-CoV-2 is unclear. Recent work by [22] analyzed the relationship between COVID-19 and meteorology and concluded that ambient temperature, relative humidity, and wind speed do modulate SARS-CoV-2 transmission, but no precise mechanism was identified.

Moreover, there is plenty of scope for disagreement in this intricate relationship [23]. Relationships were mostly place and facility-specific [24]. Among these environmental research fields, the wind environment is a rather complicated branch which is an important factor that affects virus transmission [25]. A study done in Iran found that outbreaks at low wind speed were significant [26]. Another study carried out in Turkey observed that wind speed is positively related to the number of cases [27]. In contrast, a study conducted in China revealed that the higher rate of COVID-19 was significantly associated with higher wind speed. Higher risk of the disease therefore correlated with the increasing wind speed [28]. Meanwhile, some studies related the wind environment indicated that wind speed and atmospheric stability (turbulence intensity) were closely associated with the spread of COVID-19 in the macroscale or mesoscale environment [29,30,31]. However, the influence of wind conditions does seem to be a major and complex component, but so far, very few studies have been done, especially in the relatively small-scale space.

SARS-CoV-2 is a pathogen that has recently jumped from animals to humans [32], even worse, it causes COVID-19, mainly spreads from person to person. People can be infected by a variety of routes other than larger droplets from sneezes and coughs, and there is direct evidence that smaller particles called aerosols can be spread further and wider [33]. It is noted that thousands of microdroplets per cubic centimeter can be released by a coughing, with the droplet concentration increasing with the cough flow rate [34]. Moreover, regular respiration also leads to microdroplet production attributed to fluid film rupture in the respiratory bronchioles during inhalation leading to the formation of droplets released during exhalation [35]. Furthermore, a substantial proportion of particles vented from coughing and regular breathing will hang in the air for many hours in turbulence [36]. Wind may have potential for maintaining the transmission cycle and threaten to lead to further spread, thereby reaching a larger scale.

### 2.2. Train-Induced Wind

As shown in Figure 1, a vehicle’s movement causes deformation in the surrounding air, creating wind effects induced by trains [37]. The blast blown by a train running at a high-speed creates a strong wind that affects the surrounding environment and people, and the wind is turbulent. People waiting on platforms near the running train can be discomforted or in danger [38]. The wind also can blow dust, snow, and debris towards people, and loose items or equipment on the platform can be lifted or thrown about. It has happened before that fatalities have occurred when child strollers were blown into the side of the train [39].

Each train-induced wind has unique traits owing to differences in the train’s type [40,41], not just train speed. The majority of serious incidents recorded in Europe were caused by freight trains despite running at a slower speed than HSTs [42]. However, more detail about how the effect of train-induced wind interacts with COVID-19 spread need to be elaborated. Thus, as non-stopping HSTs pass crowds, it is crucial for safety, as a potential risk, to address the problem of preventing and controlling pandemic viruses such as SARS-CoV-2.

The full-scale field test is a reliable method for measuring train-induced wind [43,44]. Nevertheless, in fact, for the full-scale field test, some challenges need to be faced, such as expensive and challenging railway vehicle authorization, requiring many on-track tests and time, as well as measurement distance. Furthermore, the results are difficult to shield from natural wind due to the extremely sensitive environmental conditions, and full-scale tests intervene too late in the development process to significantly impact design [45]. In order to find more authentic similarities, numerous attempts have been made in research methods to achieve the requirements of train-induced wind reconstruction. Model tests recently came into being because of their low cost and simplicity compared to full-scale field measurements. Therefore, moving model tests are gradually becoming the principal reference system because the train model that runs along a track can more accurately simulate the relative movement between the train, the ground, and the surroundings [46,47,48].

## 3. Experimental Set-Up

### 3.1. Moving Model Rig

**Figure 2:**
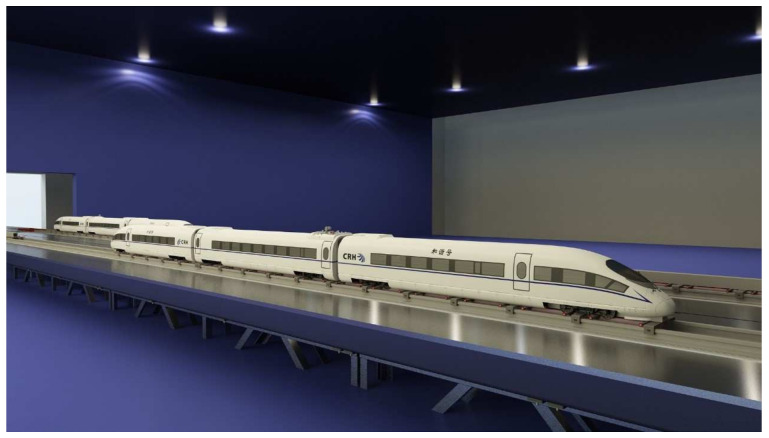
Moving model experimental set-up in CSU.

The moving model experiment was performed in the National Engineering Laboratory for High-speed Railway Construction of Central South University [49]. The moving model test rig has a 34 m long track across a test section. As shown in Figure 2, the acceleration and deceleration parts are on both sides, and the available test part is 12 m long across the test section. A 1/16.8 scaled model of a CRH—a high-speed train in operation throughout China—was used in the present experiment.

**Figure 3:**
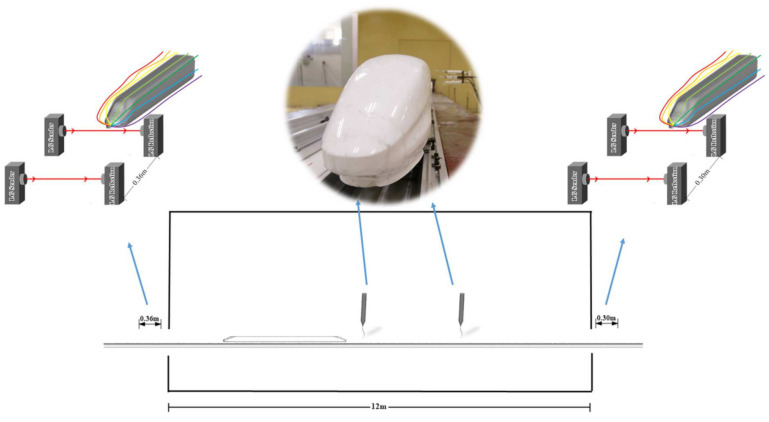
Train speed and train induced wind velocity measurement positions.

As shown in Figure 3, the length to height ratio *L/H* of the tested train model is 7.12, where *L* is the model length of 1.616 m. The *L/H* of the present tested model was smaller than that of a real train consisting of multiple carriages, limiting the boundary layer’s development along the train, but the whole variant trend is reserved. Furthermore, [48] pointed out that a small Reynolds number in the experiment can compensate for a reduced L/H effect. Thus, the effects of L/H were not considered in the present experiment. The reduced L/H has the potential to be offset by the reduced Reynolds number of the scaled model compared to full scale, as boundary layer thickness is inversely proportional to Reynolds number. In the experiment process, two pairs of photoelectric gates were installed at the test section entrance and exit to determine the model speed. Two Cobra probes measured the train-induced wind in the middle of the test section.

### 3.2. Wind Speed Measurements

Instantaneous velocities within the train-induced wind and near wake were measured using the Cobra Probe (Turbulent Flow Instrumentation P/L), a four-pressure-hole probe with a high-frequency response capable of measuring velocities in three directions [49]. 

**Figure 4:**
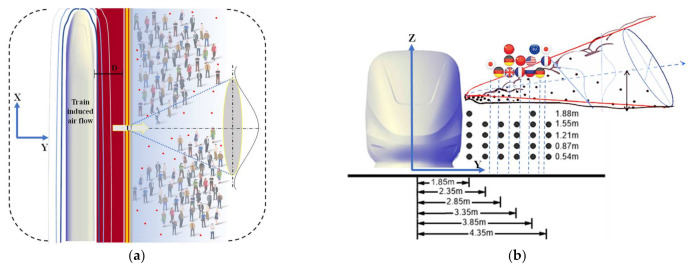
(**a**) Virus spread by train-induced wind and definition sketch; (**b**) Monitor points for train-induced wind velocity around the train (flag icons: minimum safe distances from passing trains for different countries).

To get the comprehensive understanding of the train-induced wind, speeds were measured on the train side with a series of monitoring points, as shown in Figure 4. Although some countries have yellow lines as shown in Figure 4a, which restrict walking in access to certain zones, regulations are not the same in various countries. There are still many countries and areas that do not have standards or regulations to design the relative aerodynamic effects for safe distances and disease control between people and passing trains. Due to the fact that the train could pass through the city, market, or even somewhere that is within half a foot of walking distance to the train, except for some countries’ provisions [44,50,51,52], several measuring positions are very close to the train and have also included in the present experiment. It is worth mentioning that some measuring positions in the experiment were difficult to detect in the full-scale field test. Flag icons in Figure 4b, corresponding to the safe distance lines from major countries, were prescribed. The X-direction is aligned in travel direction, Y-direction is the horizontal lateral direction, and Z is the vertical direction. X = 0 corresponds to the tip of the model train nose. Based on the consideration of the possible locations of virus carriers, the position and height of the series of measurement points A are located at Y = 1.85 m, 2.35 m, 2.85 m, 3.35 m, 3.85 m, 4.35 m (measured from the centre of the train) and Z = 0.54 m, 0.87 m, 1.21 m, 1.55 m, 1.88 m (measured from the surface), respectively. The measurement plane was located at the center of the train model along the X-direction. For each point, the Cobra probe sampling frequency was 2500 Hz and synchronized with the signal from photoelectric gates; thus, the measured velocity could coordinate with the train model’s location.

The Cobra probe pointed relative to the -x-direction during the measurement. Thus, the measured *u*, *v*, and *w* velocity components correspond to velocities in the *X-*, *Y-*, and *Z*-directions, respectively. The X-direction velocity was u, in the Y direction was v, and in the *Z* direction was w. The resultant of u and v components of velocity (U) is presented in the results, as this ‘horizontal velocity’ presents the risk for wind-borne spread of SARS-CoV-2 to a person. This definition follows TSI regulation [52] and EN [44]. This horizontal velocity is herein referred to as the ‘prevailing wind speed’: $U=\sqrt{u^{2}+v^{2}}$. Based on TSI standards, each measurement should be repeated at least 20 times to validate the result and create an ensemble-averaged result [53]. For the present experiment, the measurement at each monitoring point was repeated 25 times to get reliable ensemble averages. This uninterrupted work and these repetitions are particularly relevant to today’s train of mass transit type (i.e., over 3.6 million people passing through the busiest station daily in the world and by daily rail traffic with 100 to 180 trains per hour passing through). Therefore, the ensemble average wind speed should make the assessment more representative. Due to the wind coming from the train’s movement, all measured results were normalized by the train model speed *U_t_*. Due to the Cobra probe limitation and very high sampling frequency, in order to reduce electrical noise, a low-pass filter and a moving ensemble size at each increment in X have been applied at each measuring location. For easy comparison, the spatial coordinates were also converted into full-scale quantities. For example, L = 1.616 m of the model corresponds to 27.15 m for the full-scale train.

## 4. The Wind Speed around the High-Speed Train

**Figure 5:**
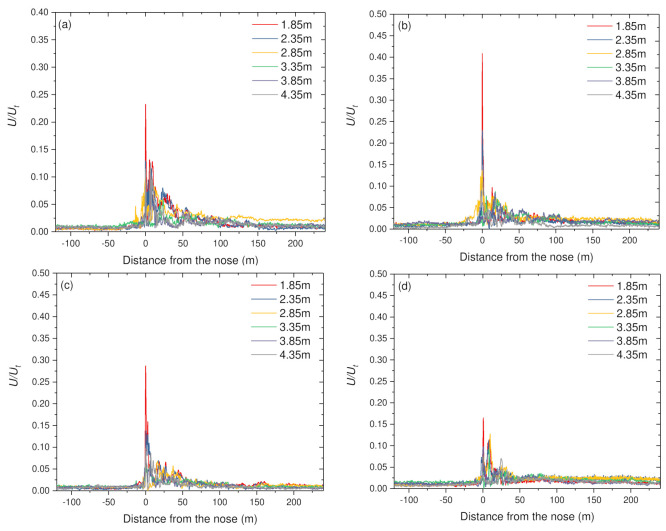

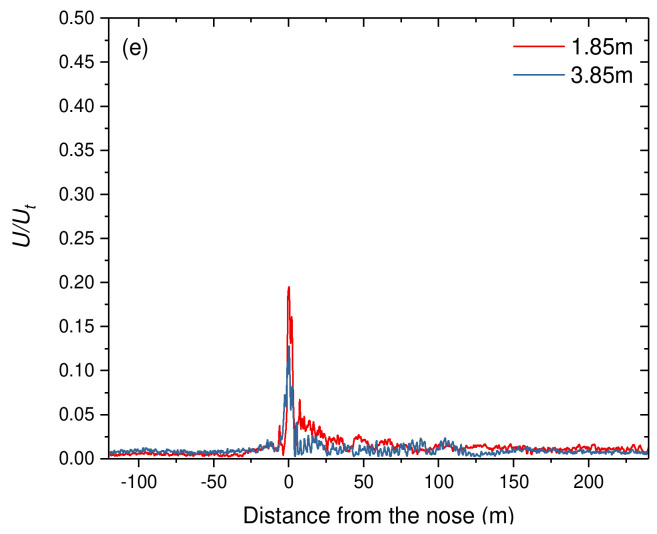
The normalised ensemble horizontal velocity: (**a**) H = 0.54 m; (**b**) H = 0.87 m; (**c**) H = 1.21 m; (**d**) H = 1.55 m; (**e**) H = 1.88 m.

In this section, data coming from all measured points are compared to the ensemble-averaged wind speed of the moving train. This section assesses the wind characteristics using data from multiple positions at a constant height and varying lateral positions, as shown in Figure 5. Generally, the trends are quite similar for the tests at a different height, especially similar velocity values, until the anemometers entered the boundary layer. The velocity changes with the developing boundary layer along the train’s length, and peaks appeared around the nose. Then, a series of violent swings appeared near the tail. After, the velocity decayed off as the probe gets farther away from the train. From the perspective of height, wind mean values for the train are initially superposed. Deviating backward from the leading nose, the wind has stable peaks, though the peak’s magnitude is variable due to different heights. The lower anemometers recorded a limited jump in the wind velocity value in the upper and lower ends of the measuring height, while the middle ones recorded a significant increase of it. Once the curves overlap again, the decay is very similar. From this point, the measurements at the lower and middle positions is higher than those measured by the upper. Then, except for the measurement at the height near the surface and rail, the behavior is similar.

Insight into the distance from the train, all measuring positions, a sharp peak of the wind can be found near the train’s head. After that, velocity reduces sharply and then is followed by a larger crest towards the tail end. The distribution of wind speed was more complicated at the lower height relative to higher heights. This observation is expected because of the complex geometry and the connection mode of wheel-rail contact under the train model as well as the track and surface. Similarly, the distribution of wind is much more complex closer to the train. In general, the wind speed decreases with increasing distance to the train, and the highest peaks are found near the track, where the flow is most influenced by the unshielded wheelsets [54].

## 5. Assessing the Extent of Wind-Borne Spread of SARS-CoV-2 near the Train

The data presented above demonstrate the winds induced by the train generated around the train and their magnitude. In order to estimate the train-induced wind as it imposes a potential risk to people’s health, the wind speed in the present work is used as inputs to a Gaussian aerosol transmission [55]. The following section presents the experiment results combined with the mathematical model, which highlights the virus-spreading implications of train-induced wind.

### 5.1. Gaussian Puff Diffusion Model and Model Implementation

Recent studies on COVID-19 have interpreted that sternutations and coughs not just consist of mucous and salivary droplets but, importantly, are primarily made of a turbulent multiphase gas (a puff) cloud that entrains surrounding air and carries aerosol particles. [56]. The Gaussian puff model affords a approach of estimating the concentration of virus downwind from a source [57]. It also takes into account transient effects and the sudden burst of the virus from an infected person. It is a method that enables the relationship between wind speeds, source strengths, and turbulent condtions to be quantified.

First of all, an orthogonal Cartesian reference system is assumed with its origin corresponding to the source’s base position and the X-axis parallel to the train travel direction, as shown in Figure 4. The Y-axis is horizontal and perpendicular to the X-axis, while the Z-axis in the vertical direction corresponds to the height from the ground direction.

Two factors need to be considered in the development of the virus in the puff models: (1) wind speed and orientation to determine variations in the position of the center of each puff, and (2) decrease of concentration around the center of the puff to determine the duration of puff. The concentration of virus in a puff for the target located downwind can be calculated using the 3D equation as shown:

(1) $$C=\frac{Q}{{8(\pi t)}^{3/2}{{(\sigma }_{x}\sigma_{y}\sigma_{z})}^{1/2}}\times exp[-\frac{{\left(x-Ut\right)}^{2}}{{4\sigma }_{x}t}-\frac{y^{2}}{{4\sigma }_{y}t}-\frac{z^{2}}{{4\sigma }_{z}t}]$$

where *C* is the virus concentration, *Q* is the source intensity; *U* is the wind speed induced by train, and *σ_x_*, *σ_y_*, and *σ_z_* are the eddy dispersion coefficients in the *x*, *y*, and *z* directions, respectively, and depend on the distance downwind.

Although *σ_x_*, *σ_y_*, and *σ_z_* have not generally been measured for lab-scale wind tunnel chambers with high-speed trains, experimental work in the context of atmospheric science provides insight on the expected behavior [58]. Due to the sufficiently turbulent in airflow such that the contribution from molecular diffusivity can be negligible, we restrict attention to short distances sufficiently close to the patient, viz.,

(2) $$\sigma_{x}{=i}_{x}y\;\sigma_{y}{=i}_{y}y\;\sigma_{z}{=i}_{z}y$$

where *i_x_* = *u_x_*/*U*, *i_y_* = *u_y_*/*U*, and *i_z_* = *u_z_*/*U*, and *ux*, *uy*, and *uz* are the RMS turbulent velocities in the *x*, *y*, and *z* directions. *U* is the ensemble average wind speed.

### 5.2. Scenario of People Standing near the Rail as the Train Pass by

**Figure 6:**
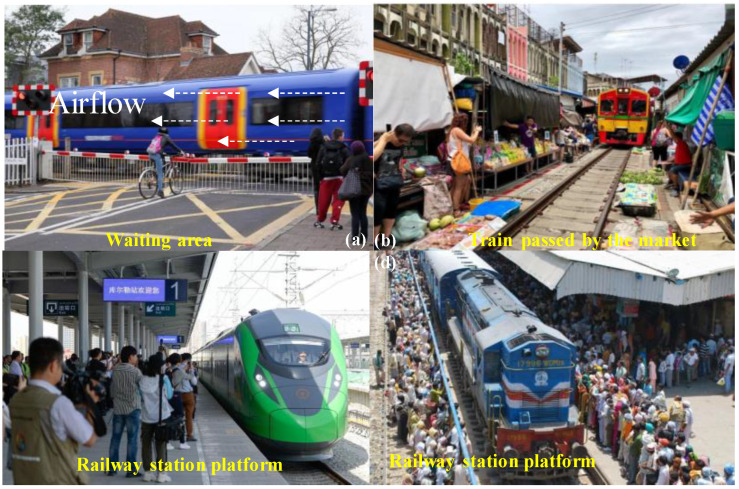
People waiting for a train running past (**a**) a crossing in the city; (**b**) market; (**c**) platform.

The assessment focused on the passengers waiting for the train, as mentioned earlier. Because people are staying near the train roaring past in many cases, potential risks need attention, as shown in Figure 6 These places always play the role of the gathering venue and public space in transportation. In assessing risk, we can look at people in two ways: potential virus producers and potential receptors. The infected cases’ spatial distribution is still uncertain since passengers can freely walk through the place, although they would be in queues or crowds according to formulary requirements. Given steady conditions, the greater the population density, the greater the risk of wind-borne spread. This study assumed one severely infected person (the highest infection number is about 654,388 in a single day to the total reported number before 20 November 2020. [59]) in the crowd waiting for the train throughout. The airflow was dominantly along with the place due to the symmetrical region. After simplification, the virus concentration was only calculated in the distance traveled (centered on the x = 0) × 2 m (centered on the measuring point and extend back) × height of measuring point.

### 5.3. Wind Condition

It was demonstrated in Section 3 that the wind speeds generated at the train’s nose subjected to break through the air resistance are maximum compared to those at the following train body. The train-induced wind is a current of air caused by the train nose and does act on the originally still-surrounding air. With the air pushed away, there is a lateral diversion for the airflow to spread out. According to Section 3, the normalized wind velocity peak is sensitive to the train’s type, and the wind speed is proportionate to the train speed in the nose region. Furthermore, the normalized wind velocity peak in the nose region of the similar streamlined trains are very close. In addition, [40] also found that the ensemble nose peak’s size is very similar values for streamlined trains in the full-scale field test, and the wake velocity could vary significantly. As the virus puffs move around the train side under the train-induced wind’s influence, passengers will suffer due to the nose peak unavoidably, as the train’s nose will cross the area where people are standing along the X direction beginning to end. Furthermore, the nose peak was chosen because in this region, some of the highest wind speed a pedestrain is liable to encounter occurs and could reduce boundary layer and shape effects to a minimum. Thus, the nose peak becomes the highly representative wind speed and the major concern of people in terms of safety and disease transmission in this study. The wind speeds selected are maximum values from samples along a line in the travel direction at the outermost scope of measuring locations (Y = 4.35 m, Z = 0.54 m, 0.87 m, 1.21 m, 1.55 m; Y = 3.85 m, Z = 1.88 m), which are either within or exceeding the respective national regulations. Wind speed close to the ground has been chosen due to possible situations because it is considered highly like a baby stroller or wheelchair or seat would be there. In a report by [59], the authors describe the latest findings that children under age five and the elderly may be at high risk from COVID-19 because of their overreacting to the viral infection through a comprehensive genotyping analysis of existing SARS-CoV-2 mutations. Also noteworthy, coronaviruses are zoonotic, which means they can be transmitted between animals and humans [60,61]. The furthest positions of wind speed sampling were chosen as Y = 4.35 and 3.85 m because these are further away from the train side and the wind speeds are relatively weak and highlight the influence of the train-induced wind. In order to establish the real rates, the measured absolute wind speeds are applied.

### 5.4. Viral Source

When considering wind-borne transmission, the number of pathogens are exhaled by the infected person. The virus are released depending on each individual’s morphology that varies from one person to another. It is a quantity that very hard to measure directly, so instead, we can obtain insight from consideration of the measured viral concentrations with the person (typically from nasal titers) [62] or assume the concentration of SARS-CoV-2 based on a similar level of MERS-CoV [63]. Based on the published studies from 30 March 2020 to 17 May 2020 [64,65,66,67,68], Ref. [62] used these data to estimate virus cumulative total emissions of up to 36,030 copies/cm^3^ per cough. Ref. [63] assume the concentration of SARS-CoV-2 with a similar level of some other Coronavirus (i.e., MERS-CoV, SARS-CoV) concentration, and find the total viral shedding virus estimations are about 3 × 10^2^ to 3 × 10^5^ copies per hour, which also agrees well with [62,65,69]. Several studies show that the viral load of SARS-CoV-2 in the lungs is higher than those of the upper airway [64,70]. Lungs give off smaller aerosolized particles in which 80–90% of droplet sizes are < 1 μm. Indeed, it is just what has been proven regarding aerosol transport of COVID-19 [71]. Infections with a higher viral load in the upper respiratory tract may be more likely to be droplet spread. Since breathing and talking occur more frequently than coughs and sneezes, the aerosol has a critical role in viral transmission, especially from asymptomatic cases [70,71].

### 5.5. Aerosol Transport

Traditionally the concentration of airborne pathogen-laden droplets indoors has been assumed to be spatially invariant in the surrounding air [72,73]. In contrast, the air exchange was dependent on natural ventilation here, and more importantly, the wind will affect and change the transmission within airflow occurring in various directions. In other words, the air is not well-mixed, and the concentration depends on the patient location along with the wind. Notably, regular breathing and talking are more significant factors than sneezing or coughing, so the contribution of high-velocity jets due to sneezing and/or coughing on the droplet transport does not need to be considered. The airflow in typical train-induced wind is turbulent, so upon exhalation, the droplets do not simply move in a straight line; the turbulent eddies work to disperse the droplets in directions orthogonal to the mean flow direction. In choosing the model, several assumptions have been made. First, we assume that all droplets impacting the ground are absorbed perfectly such that no droplets ‘bounce’ back into the airflow. Then, we assume that the environment is an open space without other structural interference (e.g., ceiling, pillars, walls) such that the viral flow is not affected by them; in other words, this approach will be valid provided that the virus’s width puff is a mere fraction throughout the environment.

### 5.6. Development and Decay

After the virus is expelled from the infected person, it goes through a slightly more complicated series of transformations. Note that COVID-19 is still under study and investigation, and under the present condition, the jury is still out. Regarding the airborne transmission of COVID-19, the World Health Organization first announced the virus’s duration to remain in the air in an isolated environment for a two hour period [74,75]. Subsequently, a series of experiments found that SARS-CoV-2, the virus that causes COVID-19, can remain infectious in the air for up to three hours [76,77]. Nevertheless, a newly published study analyzing the virus remained viable on human skin for about 9 h, nearly five times the survival time of a strain of the influenza A virus (IAV). When mixed with air pollution or settling on surfaces, the virus can survive for up to nine days [77,78,79,80,81]. As a hypothetical example, after 7 days, SARS-CoV-2 could still be found viable on the outer layer of a surgical mask or appear somewhere that people may touch since wind may increase the risk of transmission and foster the spread of COVID-19. Until now, there is no evidence that novel coronavirus vitality changes measurably on a time scale of tens of seconds. Accordingly, virus survivability issues are assumed not to apply to the short time scales of interest here. In other words, the absolute number of pathogens in any given droplet is assumed to remain constant during transport; neither any viral replication nor deactivation occurs.

### 5.7. Deposition

The concentration of airborne virus in a puff will be reduced by physical loss through the deposition. This is directly related to the vertical dispersion coefficient (*σ_z_*). If the coefficient measured from the experiment is large, the virus particles rapidly move above ground level, and so the loss from deposition is small. However, when *σ_z_* is small, more of the particles stay at ground level, and the loss rate through deposition will be greater. Additionally, it can be assumed that there is no loss of infective material. Since the probability of infection depends on the dose, and the infectious dose required to become infected with COVID-19 is unknown, there is a minute probability that a tiny dose can cause infection. A review of a wide range of respiratory viruses suggests that the infective dose is often low [62]. Because of the highly infectious nature of COVID-19, it only takes one patient to become infected for the disease to become established.

### 5.8. Result and Discussion

The experimental data presented above demonstrate the level of wind amplification that can occur around the train’s nose. In order to give a practical dimension to the results, the ensemble average nose peak velocities for 25 instantaneous velocities sampled are used as inputs to a methodology model of virus concentration. The following study presents an open space aerosol dispersion Gaussian puff model, which was developed and employed to evaluate the influence of train-induced wind on the concentration change of transferring aerosol particles from the patient to an uninfected person, highlighting the train-induced wind effect on the transmissibility of the virus. This is somewhat of an idealization, and the presence of a structure will affect the wind flow around the train. However, this work is a baseline case and is intended to provide worst-case scenarios of the wind-borne spread of the COVID-19 virus due to train-induced wind.

In this case, the infection sources at the measuring position as aforementioned. The 3D velocity field generated by the train-induced wind lead to the spread of a lot of small particles within the aerosol. This modeling aimed to estimate the aerosol spread range but not minute details of aerosol evolution on a centimeter-scale.

**Figure 7:**
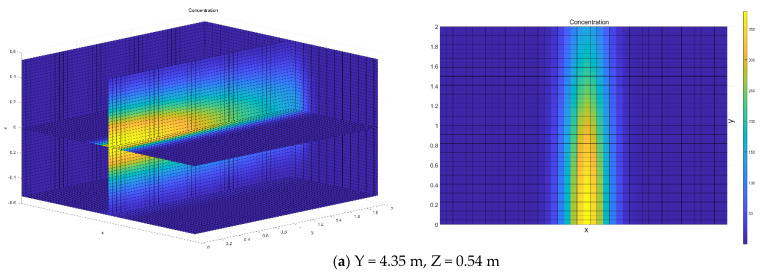

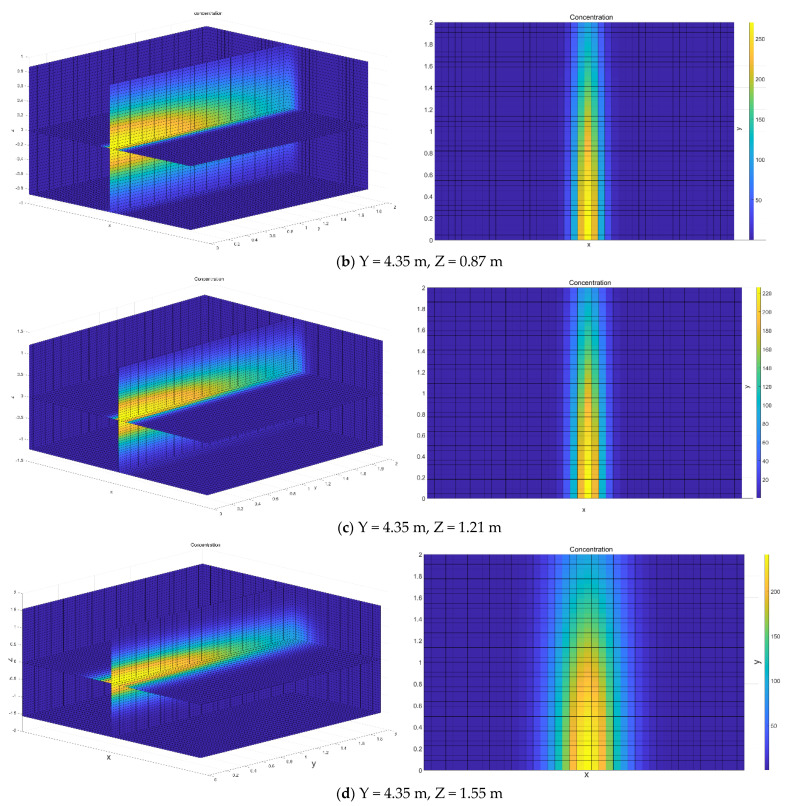

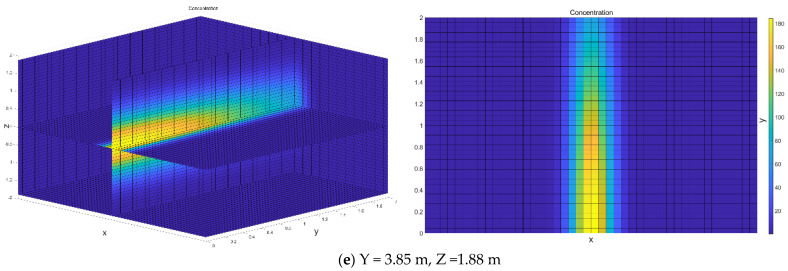
Concentration of transmission at different positions.

Figure 7 shows the effect of varying the wind speed induced by the train on the side where people are standing (Y = 4.35 m, Z = 0.54 m, 0.87 m, 1.21 m, 1.55 m; Y = 3.85 m, Z = 1.88 m) on viral concentrations. The aerosol evolution concentrations show a fast movement of the puff defined by contour plot lines. The contour plot indicates certain concentration levels. Overall, it was found that the aerosol particles concentration field forms a narrow puff from the patient towards downwind; aerosol particles were carried out with the flow and spread from the source in all directions. An open space geometry modeled with measured wind speed from experiments shows that small particles travel considerable distances, greater than 2 m. Note the strong peak at approximately X = 0, which occurs well after the train passes the receptor at t = 1 s. Qualitatively, these results are expected: the higher the wind speed, the more rapid the Y directional diffusion, so the virus will be pushed to the margins. As shown in Figure 7d, the range of horizontal diffusion is greater than that of others at a relatively low-speed point. It is noticeable that halving the wind speed from 6.24 to 2.87 m/s does not produce a doubling of concentration on the edge. This demonstrates the non-linearity inherent in the model and the nature of turbulent winds. In addition, due to the different rotational speed of the vortex, the flushing of the virus consequently varies from point to point. Even worse, 2 m social distance has apparently failed to protect the passengers for their health. The case modeled in this work is an idealized scenario and does not take into account local infrastructures such as complex terrain and buildings. Local infrastructure is likely to reduce wind speeds and create complex airflow conditions around terrain or buildings in the puff pathway. The terrain and building wake effect could generate huge turbulence and result in more deposits.

Furthermore, the train-induced puff entrainment could intensify the spread of the virus at the platform or other train side area with increased incidence and geographic expansion. It means the data presented here are a conservative estimate of risk and opens the subject of train-induced wind to further investigation. Such investigations will have more complex case set-ups, including station geometry and people in an assembled crowd. It seems almost inevitable that the risk of infections for people are higher in an assembled crowd. However, as a baseline case, the present work has shown that wind caused by the train running amplification can cause further spreading of the virus and hence poses a risk to defend and control the epidemic. Further, this study utilizes generic aerosol modeling, and we have not specified infection here, but the size distributions and concentrations of particles have been chosen to be close to these reported in the literature for SARS-CoV-2 or recent airborne viral infections close to SARS-CoV-2 (SARS, MERS). If other kinds of virus-laden aerosol particles behave similarly to aerosols described here, then the virus can be transferred over distances exceeding the currently recommended 2-m safe zone considerably.

In orbit traffic transportation, passing trains become a significant safety problem as the passing train’s speed increases. In the study of train aerodynamics, the moving train model tests are comparable with the full-scale field tests. Nonetheless, because high-speed trains run at much faster speeds in actual operation than traditional trains (i.e., the maximum speed is 350 km/h in China), the generated wind speed is extremely strong, and the effect of the train’s induced wind could be devastating. As there is no physical barrier between the rail and passenger as well as the nearby facility, in other words, passengers may stay in air thick with viral loads, and they are exposed every time they breathe in. By summarising design principles, it can be concluded that no standard or regulation was found relative to wind effects for major outbreaks of epidemics or other public health hazards in the passenger waiting area. Some countries like China, the UK, and Germany protect passengers from aerodynamic effects, while in others where safe distance lines are present, they are not based on aerodynamic safety but designed to keep the passengers from falling onto the tracks or being hit by a train. Still, these approaches do not provide information about the safe spot for epidemic prevention and control. When designing the area where trains frequently pass with severe space constraints, the existing design principles are limited.

## 6. Conclusions

SARS-CoV-2 is a highly transmissible and lethal pathogen, with more than 169 million people infected and more than 3.5 million reported deaths in the world at the time of this writing. COVID-19 represents a very high risk of infection for the general population at the global level, according to the WHO (World Health Organization, 2021). Therefore, this paper aims to analyze the influence of train-induced wind on the COVID-19 pandemic. We presented results from moving train tests of wind field around a CRH train and used these data as inputs to a Gaussian puff model to determine the virus’s concentration distributions. Using descriptive and multivariate analyses through the experimental results and theoretical model, we explored the associations between COVID-19 cases and proximity to train platforms and railway-related public transportation. Train-induced wind is an essential factor in facing the COVID-19 pandemic. Even if passengers stay far from the train, small particles can travel more than 2 m considerably. The setting of a safe distance line alone cannot prevent and control the epidemic. This study highlights the requirement for robust policies and strict respiratory protection to detected infected travelers and the need to supply a clear and safe waiting and work environment to prevent the spread of future waves of pandemic. Otherwise, transportation workers and passengers of communities near the train will be at risk for future outbreaks. As things stand, adjusting passenger’s behavior and increasing social distance should be the best option. Future studies should look at the infected population level and contact tracing to assess the risk and association between passengers, railway employees and exposure to COVID-19 in the different railway environments.
